# Structural characteristics of China's COVID-19 patent cooperation network at the province, city, and patent applicant levels

**DOI:** 10.3389/fpubh.2022.985576

**Published:** 2022-08-25

**Authors:** Wei Xia, Zilin Wang, Jindou Zhang, Jianping Yu, Liping Qiu, Zedong Yang

**Affiliations:** ^1^Institute of International Business and Economics Innovation and Governance, Shanghai University of International Business and Economics, Shanghai, China; ^2^School of Trade Negotiations, Shanghai University of International Business and Economics, Shanghai, China; ^3^School of Management, Beijing University of Chinese Medicine, Beijing, China; ^4^School of Business Administration, Jimei University, Xiamen, China; ^5^China Center for Economic Research, East China Normal University, Shanghai, China; ^6^Xinjiang Innovation Management Research Center, Xinjiang University, Urumqii, China

**Keywords:** COVID-19, patent cooperation, social network, China, structural characteristics

## Abstract

The Chinese Plan has provided an important model for the global fight against COVID-19 since its outbreak. The present study describes the structural characteristics of China's COVID-19 patent cooperation network at the province, city, and applicant levels by using social network analysis based on data from the Incopat global patent database since 2020, which helps to clarify the current technology accumulation in this field in China, and provide patent information support for the scientific efforts to fight against COVID-19. The findings are as follows: First, the inter-regional cooperation level in China's COVID-19 patent cooperation network shows a decreasing trend from eastern to central to western regions. At the inter-applicant cooperation level, kinship-based cooperation is the strongest, business-based cooperation has the widest scope, while proximity-based cooperation exists throughout these two main models of cooperation. Second, coastal provinces and cities occupy a core position in the network, and play an important role in utilizing structural holes and bridging. Patent applicants with high centrality are mostly firms. Research institutes and universities mainly play the role of bridges. Third and lastly, there is no large number of cliques at the province and city levels. However, there is a tendency for cliques to develop at the applicant level. Hence, actions are needed to prevent the development of information barriers.

## Introduction

The outbreak of Coronavirus Disease 2019 (COVID-19) at the end of 2019 has seriously threatened human health. Research teams and pharmaceutical companies around the world are stepping up to develop related products to fight against COVID-19. Meanwhile, joint R&D and patent application has become a common strategy due to the difficulty and low success rate. Governments of various countries have developed and implemented a range of measures regarding the application and protection of COVID-19 related patents. Patents have become an effective tool in the fight against COVID-19. In this context, China should pay attention to the R&D and joint patent application of COVID-19 products, so as to enhance the global confidence and ability to fight against COVID-19.

A literature review on patent cooperation reveals that numerous studies have been undertaken in terms of different disciplines and perspectives to analyze the structure, characteristics, geographical distribution, technical field distribution, and evolution, etc. of the patent cooperation network using social network analysis and other tools based on world-renowned patent databases. Ejermo and Karlsson (1) believe that the patent cooperation network often causes agglomeration, and that geographical distance has a great impact on the degree of agglomeration of the cooperation network. Schilling and Phelps (2) found that patent cooperation networks with high aggregation and short average path have stronger innovation capabilities. Fleming et al. (3) found that interregional patent cooperation networks have obvious small-world characteristics, and that patent cooperation has multiple benefits. A study of top 10 innovative countries by Ma and Lee (4) suggests that the state's support for scientific research enhances patent cooperation, whether domestically or internationally. Based on practical research in Sweden, Wilhelmsson (5) believes that patent cooperation networks mainly occur in cities with a large population and diversified industries, but the market size has a negative impact on the network, and the degree of patent cooperation in large cities is relatively low. Graf and Henning (6) found that universities and research institutes occupy an important position in the patent cooperation network constructed by cities in northeastern Germany. Goetze (7) believes that the personal network of patent inventors has a positive impact on the number of patents. Lissoni (8) found that universities that cooperate with enterprises to apply for patents are more likely to occupy the core position of university inventor cooperation networks. From the perspective of patent cooperation, Carrillo et al. (9) found that universities play the role of gatekeepers in R&D cooperation networks. Petruzzelli (10) suggests that the strength of patent cooperation between European universities and enterprises has a strong relationship with the distance between them and whether they have cooperated before. Wanzenböck and Heller-Schuh (11) analyzed the regional dynamic evolution of co-authored papers and co-patent data in Europe, and found that there are gaps in the status and power of each European region in the knowledge transfer network. Barber and Scherngell (12) found that the spatial evolution of the European patent cooperation network is heterogeneous. Lee (13) reported that the complexity of the US intercity network continued to expand and deepen during 1979–2009, and that co-invention was closely related to core urban areas. Sun et al. (14) found that the patent cooperation activities of patent applicants tend to be more frequent in the patent cooperation network in the field of new energy vehicles in China.

Research on innovation cooperation networks in the field of public health can be divided into two perspectives, which are regional and global. For example, regional research investigated innovation cooperation networks for tropical diseases, such as malaria, dengue fever, and leprosy, in Brazil (15), public health in Colombia (16), biotechnology in northeastern Brazil (17), tropical diseases in Germany (18), and health care among Spanish universities (19) to provide information for the strategic planning of health organizations in the respective regions. Global research mainly examined global research cooperation networks for a specific disease, drug, and biotechnology. For example, González-Alcaide et al. (20) analyzed the global research cooperation network for leishmaniasis to identify the roles of key countries and persons in the network. In addition, a study reviewed more than 3,000 co-authored papers on health management included in the Web of Science database in the past 13 years to identify key researchers and institutions in the cooperation networks of public health research, as well as their roles in information dissemination and health management (21). All the above studies on innovation cooperation networks in the field of public health use co-authored papers as data. When using patent data, regional studies investigated biotechnology cooperation in Brazil (22), tuberculosis prevention and treatment in Brazil (23), collaborative innovation in Chinese medicine (24), the impact of patent cooperation on firm patent output in the pharmaceutical industry in Beijing-Tianjin-Hebei region in China (25), and the impact of patent cooperation network on small and medium-sized enterprises in the pharmaceutical industry of China^1^, etc., thereby providing guidance for decision-making on regional public health development. On a global scale, Liu et al. (26) investigated the global landscape of patents related to human coronaviruses; and Zhu and Gao (27) analyzed the global biopharmaceutical patent cooperation network using a clustering comparison approach, and identified cross-border regional collaborative centers.

At present, studies on COVID-19 innovation networks mostly used co-authored papers as data sources. For example, Patil (28), Lee and Haupt (29), Yamin et al. (30), and Thavorn et al. (31) conducted bibliometric analyses of COVID-19 innovation networks based on co-authored papers and publications retrieved by the keyword “COVID-19”. However, there are few studies that examine COVID-19 innovation cooperation networks using patent data. The study by Liu et al. (26) mentioned above analyzed the global cooperation network of coronavirus patents. In particular, it not only analyzed the COVID-19 patent data, but also examined the patent data of the other six coronaviruses. Yamin et al. (30) analyzed the innovation networks in the field of emergency drugs using the patent data of COVID-19 drugs and determined its structural evolution and policy impact. Meanwhile, research on COVID-19 patent cooperation networks in China mainly focuses on drug combinations and is applied to patent design and intellectual property application (32–34). However, there are few studies that analyze network structure at the province (municipality), city, and applicant levels.

A literature review reveals that patent cooperation for COVID-19 has not been widely studied, and the structural characteristics of the COVID-19 patent cooperation network in China are more rarely analyzed. Therefore, the contribution of this paper is to explore the spatial characteristics of the fight against COVID-19 in China at the province, city, and applicant levels based on COVID-19 patent cooperation data, which will enable identification of problems from a new perspective and help to clarify the current technology accumulation in this field in China and propose optimization strategies for the COVID-19 patent cooperation network. From a practical point of view, it will hopefully provide patent information support for the scientific efforts to fight against COVID-19, such as prevention and treatment, vaccine development, and clinical drug screening. It also provides a theoretical basis for patented technology development strategies, and contributes to rethinking the geography of innovation.

## Data and methods

### Data

The data are from the Incopat global patent database. Patents with two or more applicants were retrieved from the Incopat database using keywords related to COVID-19 in the patent abstracts. The retrieval formula was as follows:

(TIAB = (xin-guan [Chinese name of COVID-19] OR xin-guan-bing-du [Chinese name of COVID-19 virus] OR xin-xing-guan-zhuang-bing-du [another Chinese name of COVID-19 virus] OR xin-xing-guan-zhuang-bing-du-fei-yan [Chinese name of Coronavirus Disease 2019] OR 2019-nCoV OR guan-zhuang-bing-du [Chinese name of coronavirus] OR SARS-CoV-2)) NOT [NO-AP = (1)].

A total of 650 COVID-19 patents were identified within the years 2020, 2021, and 2022 In the calculation of patent cooperation, for example, if a patent was applied for by three applicants A, B, and C, one cooperation between A and B, A and C, and B and C, respectively, was identified.

The retrieval was performed on April 20, 2022. Finally, a 27 × 27 matrix was formed for the inter-provincial cooperation network, and an 83 × 83 matrix for the inter-city cooperation network. For the applicant cooperation network, only patent applicants who cooperated more than five times were included, and finally a 75 × 75 matrix was formed from 696 applicants.

Three types of patent applicants are identified, which are firms, universities, and research institutes. In particular, university hospitals are identified as universities because they undertake both talent training and research. Other hospitals are identified as research institutes as they focus on research.

### Methods

The COVID-19 patent cooperation network was characterized by changes in centrality, betweenness centrality, closeness centrality, structural holes, and cohesive subgroups revealed by social network analysis.

#### Centrality

It is the main measure of the control power of nodes in the whole network (Equation 1). Normalized centrality is often used to eliminate the influence of the total number of nodes.


(1) $$\begin{array}{r} C_{D}\left(n_{i}\right)=d\left(n_{i}\right)=\underset{j}{\sum }X_{ij}=\underset{i}{\sum }X_{ji} \end{array}$$

where *C*_*D*_(*n*_*i*_) is the absolute centrality of node *i*. *X*_*ij*_ and *X*_*ji*_ are 0 or 1, indicating whether there is a relationship between nodes *i* and *j*.

#### Closeness centrality

It measures how close a node is to all other nodes, reflecting its control over other nodes.


(2) $$\begin{array}{r} CC_{i}\;\text{=}\;\left(g-1\right)/\overset{N}{\underset{j=1,j\neq i}{\sum }}d_{ij} \end{array}$$

where *g* is the number of network nodes. *d*_*ij*_ is the number of steps in the shortest path between nodes *i* and *j*.

#### Betweenness centrality

It measures the degree to which a node is located in the “middle” of other nodes, thus reflecting a state's ability to control the channels and mediate the flow of energy in the network. Assuming that the number of shortest paths between nodes *j* and *k* is *g*_*jk*_, and the number of shortest paths between nodes *j* and *k* passing through node *i* is *g*_jk_(*i*), the ability of node *i* to control the association between nodes *j* and *k* can be defined as $b_{jk}\left(i\right)=\frac{g_{jk}\left(i\right)}{g_{jk}}$.


(3) $$\begin{array}{r} BC_{i}\;\text{=}\;\frac{2\overset{n}{\underset{j}{\sum }}\overset{n}{\underset{k}{\sum }}b_{jk}\left(i\right)}{N^{2}-3N+2}\left(j\neq k\neq i\right) \end{array}$$

#### Structural holes

Effective size, efficiency, constraint, and hierarchy are the measures of structural holes. Among them, constraint is the most important measure. Therefore, only the constraint of structural holes was analyzed in this study. The operational definition of constraint is that actor *i* is indexed by the constraint of *j*.


(4) $$C_{ij}={\left(p_{ij}\;+\;\sum_{q}p_{iq}p_{qj}\right)}^{2}$$

It can be expressed as $C_{ij}=direct\;input\;\left(p_{ij}\right)+indirect\;input\;\left(\underset{q}{\sum }p_{iq}p_{qj}\right)$. Where *p*_*iq*_ is the proportion of relationships involved in *q* in the total relationships of actor *i*.

#### Cohesive subgroups

The block model approach is a way of partitioning nodes by structural information. Cohesive subgroup analysis is generally performed by convergence of iterated correlation (CONCOR). Cohesive subgroups can be used to investigate the internal association and grouping of nodes in the network.

## Topological features of China's COVID-19 patent network

### Cooperation level

**Figure 1:**
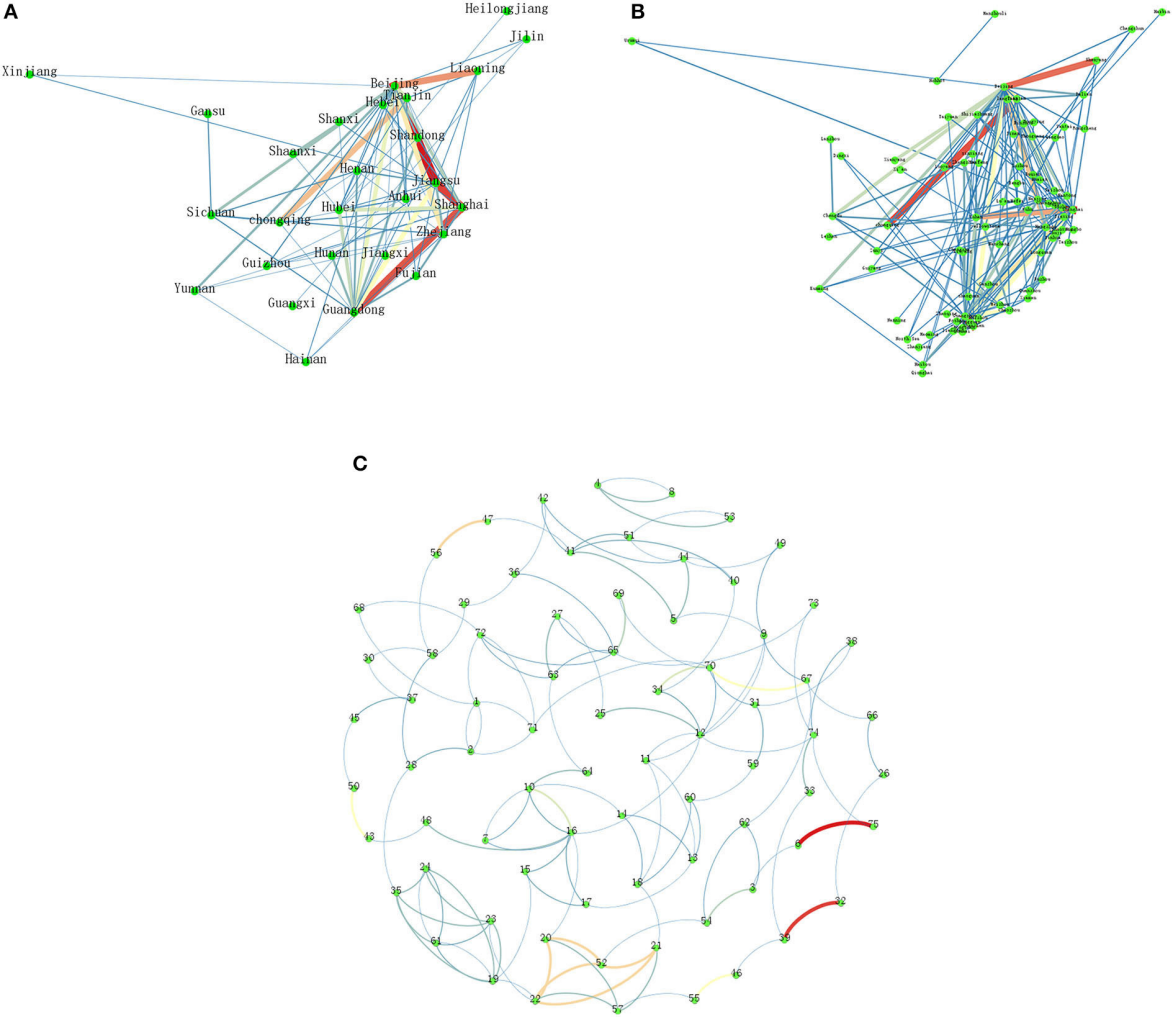
COVID-19 patent cooperation networks. **(A)** Inter-provincial COVID-19 patent cooperation network. **(B)** Inter-city COVID-19 patent cooperation network. **(C)** Inter-applicant COVID-19 patent cooperation network. Line thickness and red–yellow–green colors represent the level of cooperation. 1: Peking University; 2: Beijing University of Chemical Technology; 3: Beijing Institute of Hepatology; 4: Beijing Zhituo Vision Technology Co., Ltd.; 5: Affiliated Hospital of Binzhou Medical College; 6: Bioscience (Tianjin) Diagnostic Technology Co., Ltd.; 7: Chaozhou Kaipu Biochemical Co., Ltd.; 8: Dalian University of Technology; 9: Fudan University; 10: Guangdong Hybribio Biotech Co., Ltd.; 11: Guangzhou National Laboratory; 12: Guangzhou Institute of Respiratory Health; 13: Guangzhou Kingmed Diagnostics Group Co., Ltd.; 14: Guangzhou Kingmed Center for Clinical Laboratory Co., Ltd.; 15: Guangzhou Kaipu Biotechnology Co., Ltd.; 16: Guangzhou Kaipu Pharmaceutical Technology Co., Ltd.; 17: Guangzhou Kangjian Medical Technology Co., Ltd.; 18: Guangzhou Kingmed Translational Medicine Research Institute Co., Ltd.; 19: Guangzhou Welman New Drug R&D Co., Ltd.; 20: Guangzhou Vision Gene Tech Co., Ltd.; 21: Guangzhou Vision Medical Equipment Co., Ltd.; 22: Guangzhou Vision Medical Laboratory Co., Ltd.; 23: Guangzhou Century Clinical Research Co., Ltd.; 24: Guangzhou Xin-Chuangyi Biopharmaceutical Co., Ltd.; 25: The First Affiliated Hospital of Guangzhou Medical University (Guangzhou Respiratory Center); 26: Guangzhou Laboratory of Regenerative Medicine and Health Guangdong Province; 27: Hangzhou Medical College; 28: North China Pharmaceutical Group New Drug R&D Co., Ltd.; 29: East China University of Science and Technology; 30: Huazhong University of Science and Technology; 31: Huazhong Agricultural University; 32: Liaoning Chengda Biotechnology Co., Ltd.; 33: Southern University of Science and Technology; 34: Southern Medical University; 35: Nanjing Kangfushun Pharmaceutical Co., Ltd.; 36: Nankai University; 37: Qilu University of Technology; 38: Qingdao Marine Biomedical Research Institute Co., Ltd.; 39: Tsinghua University; 40: Qingyuan Biotechnology (Jiangsu) Co., Ltd.; 41: Qingyuan Biotechnology (Shenzhen) Co., Ltd.; 42: Qingyuanzhiguang (Wuhan) Biotechnology Co., Ltd.; 43: Sanyou Biopharmaceuticals (Shanghai) Co., Ltd.; 44: Shandong Binzhou Academy of Animal Science and Veterinary Medicine Academy Academy; 45: Shandong Modern Chinese Medicine Research Institute Co., Ltd.; 46: Affiliated Hospital of Shaanxi University of Traditional Chinese Medicine; 47: Shanghai GenePharma Co., Ltd.; 48: Shanghai Kaipu Medical Laboratory Co., Ltd.; 49: Shanghai Fosun Med-Tech Development Co., Ltd.; 50: Shanghai ZJ Bio-Tech Co., Ltd.; 51: Shenzhen Mingde Biotechnology Co., Ltd.; 52: Shenzhen Vision Medical Tech Co., Ltd.; 53: Beijing Friendship Hospital Affiliated to Capital Medical University; 54: Beijing Youan Hospital, Capital Medical AL University; 55: Beijing Hospital of Traditional Chinese Medicine Affiliated to Capital Medical University; 56: Suzhou Genepharma Co., Ltd.; 57: Vision (Shenzhen) Medical Research Center Co., Ltd.; 58: Wuhan University; 59: Wuhan Keqian Biology Co., Ltd.; 60: City University of Hong Kong Shenzhen Research Institute; 61: Xiangbei Welman Pharmaceutical Co., Ltd.; 62: The First Affiliated Hospital, Zhejiang University School of Medicine; 63: Zhejiang Pukang Biotechnology Co., Ltd.; 64: Zhengzhou Kaipu Medical Laboratory (Limited Partnership); 65: National Institute for Viral Disease Control and Prevention, China CDC; 66: Guangzhou Institute of Biomedicine and Health, Chinese Academy of Sciences; 67: Shanghai Institute of Materia Medica, Chinese Academy of Sciences; 68: Institute of Biophysics, Chinese Academy of Sciences; 69: Institute of Microbiology, Chinese Academy of Sciences; 70: Wuhan Institute of Virology, Chinese Academy of Sciences; 71: Academy of Military Medicine, Academy of Military Sciences, Chinese People's Liberation Army; 72: Institute of Laboratory Animals Science, CAMS & PUMC; 73: Wuxi Customs, People's Republic of China; 74: Sun Yat-Sen University; 75: Chongqing Medical University.

The COVID-19 patent cooperation networks at the province, city, and applicant levels within China were constructed. Results are shown in Figure 1 and Table 1.

**Table 1:** Types and levels of COVID-19 patent cooperation.

**Level**	**Cooperation type**		**Cooperation level**
Province	Eastern province	Eastern province	180
	Eastern province	Central province	86
	Eastern province	Western province	60
	Western province	Central province	7
	Central province	Central province	5
	Western province	Western province	4
City	Non-provincial capital city	Provincial capital city	221
	Provincial capital city	Provincial capital city	201
	Non-provincial capital city	Non-provincial capital city	34
Applicant	Firm	Firm	144
	University	Firm	52
	University	Research institute	33
	Research institute	Research institute	26
	Firm	Research institute	23
	University	University	16

In terms of inter-provincial cooperation, there are a total of 85 pairs of provinces/municipalities in the network. The highest cooperation level is found for Beijing–Jiangsu, which is 23, and the lowest is 1. Only 11.76% of them have a cooperation level of 10 and above, and 27.05% have a cooperation level of 5 and above. Specifically, Beijing–Jiangsu, Jiangsu–Shanghai, and Guangdong–Shanghai have the highest cooperation level of more than 20. Beijing–Liaoning, Tianjin–Chongqing, Zhejiang–Beijing, Guangdong–Jiangsu, Beijing–Guangdong, Beijing–Tianjin, and Shanghai–Hubei have the second highest cooperation level of 10 and above. Hubei–Guangdong, Beijing–Shanghai, Beijing–Shaanxi, and Sichuan–Beijing have the third highest cooperation level of 7 to 9. The remaining pairs of provinces/municipalities have a low cooperation level of below 7. In terms of eastern, central, and western provinces, the highest cooperation level is observed between eastern provinces, which is much higher than the second and third highest cooperation level that is observed between eastern and central provinces and between eastern and western provinces, respectively.

In terms of inter-city cooperation, there are 166 city pairs in the network. The highest cooperation level is found for Suzhou–Shanghai, which is 17, and the lowest is 1. Only 5.42% of them have a cooperation level of 10 and above, and 12.65% have a cooperation level of 5 and above. Specifically, Suzhou–Shanghai, Tianjin–Chongqing, and Guangzhou–Shenzhen have the highest cooperation level of more than 15. Beijing–Shenyang, Shanghai–Wuhan, Hangzhou–Beijing, Nanjing–Beijing, Beijing–Tianjin, and Guangzhou–Shanghai have the second highest cooperation level of 10 and above. Guangzhou–Chaozhou, Beijing–Suzhou, Shanghai–Shenzhen, Beijing–Shenzhen, Beijing–Xianyang, and Chengdu–Beijing have the third highest cooperation level of 7–9. The remaining pairs of cities have a low cooperation level of below 7. In terms of provincial capital and non-provincial capital cities, the lowest cooperation level is observed between non-provincial capital cities, and the highest is between provincial capital and non-provincial capital cities.

In terms of applicant cooperation, there are a total of 133 pairs of applicants in the network. The highest cooperation level is observed between Bioscience (Tianjin) Diagnostic Technology Co., Ltd.–Chongqing Medical University, which is 17, and the lowest is 1. There are only 2 pairs of applicants with a cooperation level of 10 and above, and 15 pairs with a cooperation level of 5 and above. Specifically, Bioscience (Tianjin) Diagnostic Technology Co., Ltd.–Chongqing Medical University and Tsinghua University–Liaoning Chengda Biotechnology Co., Ltd. have the highest cooperation level of more than 10. Suzhou Genepharma Co., Ltd.–Shanghai GenePharma Co., Ltd., Guangzhou Vision Gene Tech Co., Ltd.–Guangzhou Vision Medical Laboratory Co., Ltd., Guangzhou Vision Gene Tech Co., Ltd.–Shenzhen Vision Medical Tech Co., Ltd., Guangzhou Vision Medical Equipment Co., Ltd.–Guangzhou Vision Medical Laboratory Co., Ltd., Guangzhou Vision Medical Equipment Co., Ltd.–Shenzhen Vision Medical Tech Co., Ltd., and Guangzhou Vision Medical Laboratory Co., Ltd.–Shenzhen Vision Medical Tech Co., Ltd. have the second highest cooperation level of 8 and above. Beijing Hospital of Traditional Chinese Medicine Affiliated to Capital Medical University–Affiliated Hospital of Shaanxi University of Traditional Chinese Medicine, Sanyou Biopharmaceuticals Co., Ltd.–Shanghai ZJ Bio-Tech Co., Ltd., Shanghai Institute of Materia Medica, Chinese Academy of Sciences–Wuhan Institute of Virology, Chinese Academy of Sciences, Southern Medical University–Wuhan Institute of Virology, Chinese Academy of Sciences, Guangdong Hybribio Biotech Co., Ltd.–Guangzhou Technology Ltd., Institute of Microbiology, Chinese Academy of Sciences–National Institute for Viral Disease Control and Prevention, China CDC, and Beijing Youan Hospital, Capital Medical AL University–Beijing Institute of Hepatology have the third highest cooperation level of 5 to 7. The remaining pairs of patent applicants have a low cooperation level of below 5. In terms of the types of patent applicants, the highest cooperation level is observed between firms, which is 144, nearly three times of the second highest level that is observed between universities and firms; and the lowest cooperation level is observed between universities.

**Figure 2:**
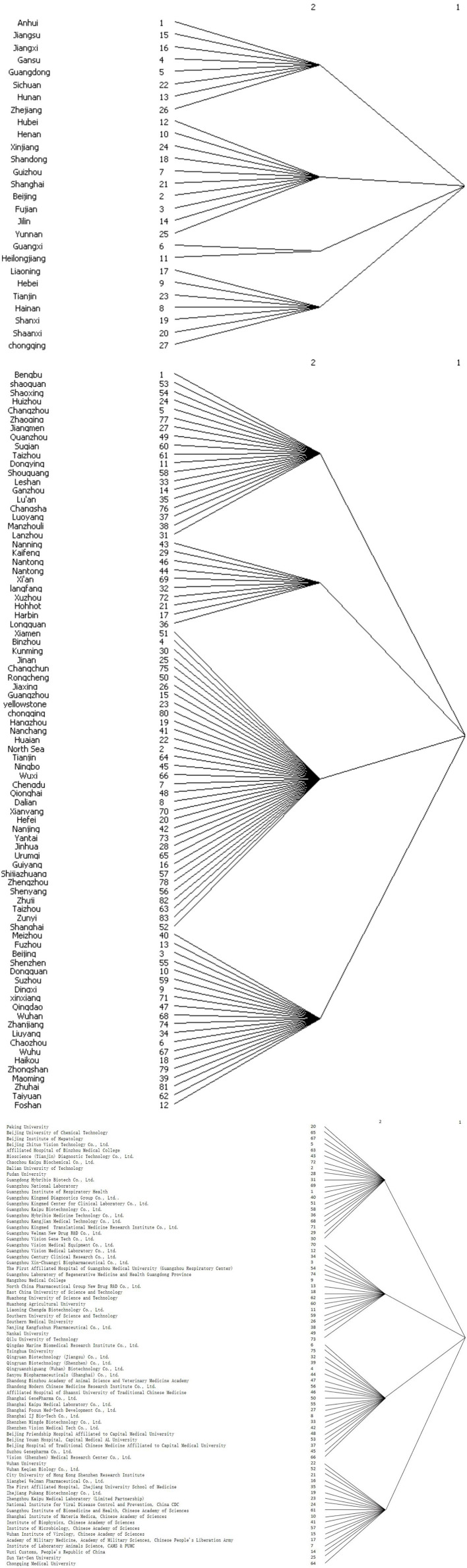
Cohesive subgroups in the COVID-s19 patent cooperation networks.

The cooperation networks between provinces and cities are mainly formed through hierarchical and contagious diffusion. The cooperation between patent applicants is mainly based on kinship and business, supplemented by geographical proximity. As can be seen from Figure 2, China's COVID-19 patent cooperation network roughly has a structure centered on the Beijing-Tianjin-Hebei Region, Yangtze River Delta, and Pearl River Delta. It suggests that the three major urban agglomerations have a high integration of the COVID-19 industry, and have high cooperation between provinces and cities. There are also some nodes in the northeastern and western regions. The north–south cooperation is stronger higher than the east–west cooperation. Developed provinces and cities have close cooperation with each other. For example, Beijing–Jiangsu, Jiangsu–Shanghai, Guangdong–Shanghai, Suzhou–Shanghai, Tianjin–Chongqing, and Guangzhou–Shenzhen have a cooperation level of more than 15. In addition, provinces and cities in close proximity also have a high level of cooperation. For example, Jiangsu–Shanghai and Suzhou–Shanghai have a cooperation level of more than 15. It suggests that geographical proximity enables convenient communication and management, which has a great impact on COVID-19 patent cooperation. Among patent applicants with a relatively high cooperation level, a business-based cooperation model between applicants with similar or the same business scope is noted at the highest level, a kinship-based cooperation model between applicants in investment relationships at the second highest level, and a business-based cooperation model again at the third highest level. However, most patent applicants are also located relatively close together in these two main cooperation models.

On the whole, the inter-regional cooperation level in China's COVID-19 patent cooperation network shows a decreasing trend from eastern to central to western regions. Eastern provinces and cities have stable partners; collaborative innovation in central provinces and cities need to be further improved; whereas there is still a lot of room for development in the western provinces and cities in establishing patent cooperation with other regions. At the applicant level, kinship-based cooperation is the strongest, business-based cooperation has the widest scope, while proximity-based cooperation exists throughout these two main models of cooperation.

### Centrality

Node centrality measures the number of nodes directly connected to a given node, reflecting the importance of that role in the network. Intuitively, a node directly connected to many nodes has a high degree centrality, which reflects its relative importance in China's COVID-19 patent cooperation network.

As shown in Table 2, in terms of inter-provincial cooperation, Beijing has the highest centrality, indicating that it occupies a central position in the network and has a strong influence on other provinces in the entire network. The other provinces, such as Guangdong, Shanghai, and Jiangsu, have similar centrality in the network, while Yunnan, Anhui, and Hunan are at the edge. In terms of inter-city cooperation, Beijing still has the highest centrality, which is far higher than the second highest centrality of Guangzhou. It indicates that Beijing has an absolutely core influence in the entire network. It carried out 111 patent cooperation activities with other cities, far exceeding that by other cities. In terms of inter-applicant cooperation, most of the top 20 applicants are firms, and the top four applicants are Vision and its subsidiaries. Specifically, Guangzhou Vision Medical Laboratory Co., Ltd. had the most relations and carried out 28 patent cooperation activities with other entities. Among the research institutes, Wuhan Institute of Virology, Chinese Academy of Sciences has the largest centrality of 17. Among the universities, Chongqing Medical University has the largest centrality. It indicates that these patent applicants have the largest number of cooperators, form a large patent cooperation network by themselves, and have access to abundant resources. Compared with other nodes, they are more embedded and occupy an important position in the network, and have a greater influence.

**Table 2:** Centrality of top 20 nodes in the COVID-19 patent cooperation network.

**Province/ municipality**	**Centrality**	**City**	**Centrality**	**Applicant**	**Centrality**
Beijing	93	Beijing	111	Guangzhou Vision Medical Laboratory Co., Ltd.	28
Guangdong	65	Guangzhou	75	Shenzhen Vision Medical Tech Co., Ltd.	24
Shanghai	60	Shanghai	66	Guangzhou Vision Medical Equipment Co., Ltd.	20
Jiangsu	55	Shenzhen	46	Guangzhou Vision Gene Tech Co., Ltd.	20
Zhejiang	36	Nanjing	36	Guangzhou Technology Ltd.	18
Tianjin	29	Wuhan	32	Wuhan Institute of Virology, Chinese Academy of Sciences	17
Hubei	29	Tianjin	32	Nanjing Kangfushun Pharmaceutical Co., Ltd.	16
Liaoning	23	Hangzhou	29	Guangzhou Welman New Drug R&D Co Ltd.	15
Chongqing	22	Suzhou	25	Guangzhou Century Clinical Research Co., Ltd.	15
Sichuan	16	Chongqing	21	Guangzhou Xin-Chuangyi Biopharmaceutical Co., Ltd.	15
Henan	15	Chengdu	18	National Institute for Viral Disease Control and Prevention, China CDC	14
Fujian	15	Shenyang	17	Bioscience (Tianjin) Diagnostic Technology Co., Ltd.	13
Hebei	13	Chaozhou	17	Chongqing Medical University	13
Shandong	12	Zhengzhou	15	Xiangbei Welman Pharmaceutical Co., Ltd.	13
Yunnan	9	Shijiazhuang	15	Qingyuan Biotechnology (Shenzhen) Co., Ltd.	12
Anhui	8	Xiamen	14	Liaoning Chengda Biotechnology Co., Ltd.	12
Shaanxi	8	Kunming	9	Vision (Shenzhen) Medical Research Center Co., Ltd.	12
Hunan	7	Fuzhou	8	Guangzhou Institute of Respiratory Health	12
Guizhou	6	Haikou	8	Tsinghua University	12
Hainan	5	Hefei	8	Guangdong Hybribio Biotech Co., Ltd.	12

### Betweenness centrality

Betweenness centrality measures whether a node occupies an intermediate position between other nodes. The higher the betweenness centrality, the stronger its control over the path. It reflects the degree of control this node has over resources, that is, the degree to which it acts as a bridge (i.e., a line of communication between nodes). As shown in Table 3, Beijing ranks first in terms of betweenness centrality at both province and city levels, being much higher than the second highest ones. It indicates that Beijing has absolute control over the path of the COVID-19 patent cooperation network, occupies a pivotal position in this network, and plays a significant bridging role in the cooperation. At the province level, cooperation in the network mainly occurs through Beijing, Guangdong, Hubei, and Shanghai. At the city level, cooperation mainly occurs through Beijing, Guangzhou, Shanghai, and Shenzhen. At the applicant level, cooperation mainly occurs through Guangzhou Institute of Respiratory Health, Guangzhou National Laboratory, Wuhan Institute of Virology, Chinese Academy of Sciences, Shanghai Institute of Materia Medica, Chinese Academy of Sciences, and Peking University. It indicates that these institutes or universities have relatively more information on COVID-19, and have control over the path of the COVID-19 patent cooperation network.

**Table 3:** Betweenness centrality of top 20 nodes in the COVID-19 patent cooperation network.

**Province/ municipality**	**Betweenness centrality**	**City**	**Betweenness centrality**	**Applicant**	**Betweenness centrality**
Beijing	36.813	Beijing	40.011	Guangzhou Institute of Respiratory Health	4.369
Guangdong	15.226	Guangzhou	22.727	Guangzhou National Laboratory	2.234
Hubei	10.213	Shanghai	11.853	Wuhan Institute of Virology, Chinese Academy of Sciences	2.147
Shanghai	8.233	Shenzhen	10.362	Wuhan Institute of Virology, Chinese Academy of Sciences	1.888
Sichuan	7.754	Nanjing	8.785	Peking University	1.777
Jilin	7.692	Hangzhou	8.293	Institute of Laboratory Animals Science, CAMS & PUMC	1.777
Jiangsu	6.885	Wuhan	6.395	National Institute for Viral Disease Control and Prevention, China CDC	1.499
Zhejiang	4.656	Hefei	4.487	Beijing University of Chemical Technology	1.481
Liaoning	1.844	Chongqing	3.267	Fudan University	1.407
Chongqing	0.972	Tianjin	2.878	Sun Yat-sen University	1.296
Tianjin	0.938	Shijiazhuang	2.481	The First Affiliated Hospital, Zhejiang University School of Medicine	1.259
Henan	0.892	Chengdu	2.359	North China Pharmaceutical Group New Drug R&D Co., Ltd.	1.222
Hebei	0.203	Chaozhou	2.326	Wuhan University	0.926
Anhui	0.141	Shenyang	2.258	Huazhong Agricultural University	0.666
Fujian	0.000	Jiaxing	2.258	Guangzhou Kingmed Diagnostics Group Co., Ltd.	0.611
Shandong	0.000	Changchun	2.258	Guangzhou Kingmed Translational Medicine Research Institute Co., Ltd.	0.611
Guizhou	0.000	Xiamen	1.633	Guangzhou Technology Ltd.	0.407
Hainan	0.000	Suzhou	0.901	Guangdong Hybribio Biotech Co., Ltd.	0.185
Yunnan	0.000	Fuzhou	0.833	Zhejiang Pukang Biotechnology Co., Ltd.	0.167
Jiangxi	0.000	Wuhu	0.600	Qingyuan Biotechnology (Shenzhen) Co., Ltd.	0.111

### Closeness centrality

Closeness centrality measures how close a given node is to other nodes in the network. It is defined as the reciprocal of the sum of the shortest path distances from that node to all other nodes. The closer a node is to other nodes, the greater its closeness centrality. The shorter the sum of the shortest paths from a node to all other nodes, the easier it is for information resources in the network to reach this node.

As shown in Table 4, at the province level, coastal provinces/municipalities, such as Beijing, Guangdong, Shanghai, Jiangsu, and Zhejiang, have higher closeness centrality. At the city level, coastal cities, such as Beijing, Guangzhou, Shanghai, Nanjing, and Shenzhen, rank high in closeness centrality. At the applicant level, universities and research institutes, such as Guangzhou Institute of Respiratory Health, Wuhan Institute of Virology, Chinese Academy of Sciences, Fudan University, Guangzhou National Laboratory, and Southern Medical University, have higher closeness centrality, occupying a prominent position in the network. It suggests that these nodes are closer to other innovative entities in China's COVID-19 patent cooperation network. The shorter path length also enables these nodes to acquire network resources or search for partners in a shorter time and with higher efficiency than other nodes.

**Table 4:** Closeness centrality of top 20 nodes in the COVID-19 patent cooperation network.

**Province/ municipality**	**Closeness centrality**	**City**	**Closeness centrality**	**Applicant**	**Closeness centrality**
Beijing	83.871	Beijing	13.399	Guangzhou Institute of Respiratory Health	1.777
Guangdong	70.270	Guangzhou	13.099	Wuhan Institute of Virology, Chinese Academy of Sciences	1.775
Shanghai	65.000	Shanghai	12.975	Fudan University	1.773
Jiangsu	63.415	Nanjing	12.693	Guangzhou National Laboratory	1.772
Zhejiang	61.905	Shenzhen	12.674	Southern Medical University	1.772
Hubei	60.465	Hangzhou	12.615	Wuhan Institute of Virology, Chinese Academy of Sciences	1.771
Tianjin	56.522	Wuhan	12.596	Sun Yat-Sen University	1.771
Henan	56.522	Shijiazhuang	12.557	The First Affiliated Hospital of Guangzhou Medical University (Guangzhou Respiratory Center)	1.769
Hebei	55.319	Suzhou	12.500	Huazhong Agricultural University	1.768
Chongqing	55.319	Xiamen	12.462	Guangzhou Kingmed Diagnostics Group Co., Ltd.	1.767
Liaoning	54.167	Chongqing	12.349	Guangzhou Kingmed Translational Medicine Research Institute Co., Ltd.	1.767
Sichuan	54.167	Tianjin	12.312	Shanghai Fosun Med-Tech Development Co., Ltd.	1.766
Anhui	53.061	Chengdu	12.312	The First Affiliated Hospital, Zhejiang University School of Medicine	1.765
Fujian	52.000	Zhengzhou	12.312	Southern University of Science and Technology	1.763
Shandong	52.000	Chaozhou	12.294	Qingdao Marine Biomedical Research Institute Co., Ltd.	1.763
Guizhou	50.980	Fuzhou	12.275	Guangzhou Kingmed Center for Clinical Laboratory Co., Ltd.	1.760
Hainan	50.000	Zhuhai	12.257	City University of Hong Kong Shenzhen Research Institute	1.760
Yunnan	50.000	Qingdao	12.221	Wuhan Keqian Biology Co., Ltd.	1.760
Jilin	49.057	Shenyang	12.184	Beijing Institute of Hepatology	1.758
Jiangxi	49.057	Haikou	12.148	Beijing Youan Hospital, Capital Medical AL University	1.758

### Structural holes

Structural holes are often used to reveal the degree of homogeneous competition among nodes in a patent cooperation network. Effective size, efficiency, and constraint were calculated using UCINET. Among them, efficiency and constraint are the two most important measures. A node with higher efficiency and smaller constraint has greater structural holes and obvious advantages. As shown in Table 5, at the province level, Beijing, Guangdong, Shanghai, Jiangsu, Hubei, Zhejiang, and Tianjin have a significantly larger effective size and efficiency and smaller constraint than other provinces/municipalities. It indicates that these provinces/municipalities are less constrained by others, have more opportunities for competition, and have significant advantages of structural holes. At the city level, Beijing, Guangzhou, Shanghai, Shenzhen, Nanjing, and Hangzhou have a relatively larger effective size and efficiency and smaller constraint. It indicates that these cities are less constrained by other cities and occupy the position of structural holes in the network. At the applicant level, research institutes and firms make greater use of structural holes. Specifically, Guangzhou Institute of Respiratory Health, National Institute for Viral Disease Control and Prevention, China CDC, Guangzhou Technology Ltd., Shandong Binzhou Academy of Animal Science and Veterinary Medicine Academy, and Qingyuan Biotechnology (Shenzhen) Co., Ltd. occupy the position of structural holes, have significant competitive advantages, and are less constrained by other applicants.

**Table 5:** Structural holes of top 20 nodes in the COVID-19 patent cooperation network.

**Province/ municipality**	**Effective** **size**	**Efficiency**	**Constraint**	**City**	**Effective** **size**	**Efficiency**	**Constraint**	**Applicant**	**Effective** **size**	**Efficiency**	**Constraint**
Beijing	18.089	0.861	0.218	Beijing	35.619	0.937	0.121	Guangzhou Institute of Respiratory Health	5.583	0.931	0.384
Guangdong	12.031	0.752	0.366	Guangzhou	25.164	0.899	0.207	National Institute for Viral Disease Control and Prevention, China CDC	5.500	0.917	0.327
Shanghai	10.717	0.766	0.423	Shanghai	20.170	0.877	0.260	Guangzhou Technology Ltd.	4.265	0.853	0.385
Jiangsu	8.346	0.642	0.469	Shenzhen	12.224	0.815	0.348	Shandong Binzhou Academy of Animal Science and Veterinary Medicine Academy	4.000	1.000	0.344
Hubei	7.978	0.725	0.542	Nanjing	11.104	0.793	0.371	Qingyuan Biotechnology (Shenzhen) Co., Ltd.	4.000	1.000	0.264
Zhejiang	7.494	0.624	0.529	Hangzhou	10.916	0.780	0.426	Wuhan Institute of Virology, Chinese Academy of Sciences	3.588	0.897	0.418
Tianjin	6.245	0.781	0.469	Wuhan	9.141	0.762	0.434	Institute of Laboratory Animals Science, CAMS & PUMC	3.000	0.750	0.625
Chongqing	4.976	0.711	0.675	Tianjin	7.970	0.797	0.407	Peking University	3.000	0.750	0.563
Henan	4.517	0.645	0.662	Shijiazhuang	6.143	0.683	0.513	Wuhan Institute of Virology, Chinese Academy of Sciences	3.000	1.000	0.540
Hebei	4.168	0.595	0.630	Xiamen	5.678	0.710	0.474	Sun Yat-Sen University	3.000	1.000	0.429
Sichuan	4.053	0.675	0.625	Suzhou	5.462	0.607	0.570	Wuhan University	3.000	1.000	0.375
Anhui	3.262	0.652	0.767	Chongqing	5.450	0.779	0.672	Fudan University	3.000	1.000	0.360
Fujian	2.903	0.581	0.728	Hefei	4.400	0.880	0.474	Guangzhou Kingmed Diagnostics Group Co., Ltd.	3.000	1.000	0.360
Liaoning	2.511	0.419	0.940	Haikou	4.258	0.852	0.510	Guangzhou Kingmed Translational Medicine Research Institute Co., Ltd.	3.000	1.000	0.360
Jilin	2.250	0.750	1.091	Chengdu	4.215	0.703	0.553	Guangzhou National Laboratory	3.000	1.000	0.333
Hainan	2.116	0.529	1.061	Chaozhou	3.790	0.632	0.601	Beijing University of Chemical Technology	2.600	0.867	0.540
Guizhou	2.035	0.509	1.009	Fuzhou	3.580	0.716	0.636	Guangdong Hybribio Biotech Co., Ltd.	2.370	0.790	0.574
Shandong	1.875	0.469	0.980	Dalian	3.303	0.661	0.885	The First Affiliated Hospital, Zhejiang University School of Medicine	2.200	0.733	0.980
Yunnan	1.580	0.395	1.084	Jiaxing	3.108	0.777	0.830	Guangzhou Vision Medical Laboratory Co., Ltd.	2.143	0.536	0.698
Shaanxi	1.107	0.554	0.934	Nantong	3.000	1.000	0.429	Qingyuan Biotechnology (Jiangsu) Co., Ltd.	2.000	1.000	0.625

### Cohesive subgroups

Cohesive subgroups are used to analyze the cliques in a group network and clique composition by quantifying group cohesiveness, thereby describing the group relationships. The external-internal (E-I) index is used to measure the dominance of external relations over internal relations, and analyze the relationships between and within subgroups in network structure analysis. It is calculated as follows:


(5) $$\begin{array}{r} E\;-\;I=\;\frac{EL-IL}{EL+IL} \end{array}$$

where EL is the number of relationships between subgroups, and IL is the number of relationships within subgroups. The value range of the E-I index is [−1, +1]. The closer the value is to 1, the more likely the relationships occur outside the subgroups, and the smaller the number of cliques On the contrary, the closer the value is to −1, the more likely the relationships occur within the subgroups, and the larger the number of cliques. A value equal to 0 indicates that the relationships in the network are randomly distributed.

The cohesive subgroups of the COVID-19 patent cooperation network were analyzed using UCINET (Figure 2). There are four subgroups in the COVID-19 patent cooperation network at the province, city, and applicant levels, respectively. As revealed by the macro structure of the network and the E-I index analysis results in Table 6, at the provincial level, the first subgroup is centered on Guangdong and Jiangsu and radiated to Anhui, Jiangxi and other provinces; the second subgroup is centered on Beijing and Shanghai and extended to Hubei, Henan, Xinjiang and other provinces; the third subgroup consists of Guangxi and Heilongjiang; and the fourth subgroup is centered on Tianjin and extended to Hainan, Shanxi, Shaanxi and other provinces.

**Table 6:** E-I index analysis of the COVID-19 patent cooperation networks.

	**Subgroup 1**	**Subgroup 2**	**Subgroup 3**	**Subgroup 4**	**Entire network**
**Province**					
Number of internal relationships	20	32	0	6	58
Number of external relationships	37	43	2	30	112
E-I index	0.298	0.147	1.000	0.667	0.528
**City**					
Number of internal relationships	4	0	86	28	118
Number of external relationships	16	13	96	89	214
E-I index	0.600	1.000	0.055	0.521	0.544
**Applicant**					
Number of internal relationships	34	32	18	52	136
Number of external relationships	14	9	9	2	34
E-I index	−0.417	−0.561	−0.333	−0.926	−0.559

At the city level, the first subgroup consists of Bengbu, Changzhou, Dongying, Ganzhou and other cities; the second subgroup consists of Harbin, Hohhot, Kaifeng, Langfang and other cities; the third subgroup is centered on Guangzhou and Shanghai, and radiated to Nanchang, Nanjing, Ningbo, Xiamen and other cities; and the fourth subgroup is centered on Beijing and extended to Chaozhou, Dingxi, Dongguan, Foshan and other cities.

At the applicant level, the first subgroup is centered on Guangzhou Vision Gene Tech Co., Ltd. and radiated to Peking University, Beijing University of Chemical Technology and other applicants; the second subgroup is centered on the Wuhan Institute of Virology, Chinese Academy of Sciences and extended to Beijing Institute of Hepatology, Fudan University and other applicants; the third subgroup is centered on Bioscience (Tianjin) Diagnostic Technology Co., Ltd. and Chongqing Medical University, and extended to Dalian University of Technology, Hangzhou Medical College and other applicants; and the fourth subgroup is centered on Guangzhou Vision Medical Laboratory Co., Ltd. and Shenzhen Vision Medical Tech Co., Ltd., and extended to Guangzhou Vision Medical Equipment Co., Ltd., Guangzhou Technology Ltd. and other applicants.

At the province and city levels, the E-I indices is positive for all subgroups. The number of external relationships of subgroup nodes is significantly greater than that of internal relationships. There is no large number of cliques, indicating a great potential for development. Nodes in the third subgroup in the inter-provincial cooperation network and the second subgroup in the inter-city cooperation network are relatively dispersive, have weak cooperation relations, and are less involved in the COVID-19 patent cooperation with other nodes. These nodes have strong external relationships. Their level of patent cooperation lags behind the overall average. They are at the edge of the COVID-19 patent cooperation network. At the applicant level, the E-I index is negative for all subgroups. The number of external relationships of subgroup nodes is significantly smaller than that of internal relationships, indicating a tendency of clique formation. Cooperation in the first to third subgroups is mainly based on business and geographical proximity, and that in the fourth subgroup is based on kinship and geographical proximity. These subgroup cooperation models hinder the flow of resources and factors within the subgroups and reduce the efficiency of patent cooperation and communication. To avoid a great number of cliques and consequent information barriers, which would restrict the overall development of the subgroups, subgroups nodes in the patent applicant cooperation should strengthen communication with external nodes and develop a diversified patent cooperation network.

## Conclusions and policy implications

### Conclusions

The present study describes the structural characteristics of China's COVID-19 patent cooperation network at the province, city, and applicant levels by using social network analysis based on data from the Incopat global patent database since 2020. The following conclusions are drawn:

The cooperation networks between developed provinces and cities are mainly formed through hierarchical and contagious diffusion. The three major urban agglomerations in China, i.e., the Beijing-Tianjin-Hebei Region, Yangtze River Delta, and Pearl River Delta, have a high integration of the COVID-19 industry. The cooperation between patent applicants is mainly based on kinship and business, supplemented by geographical proximity; specifically, kinship-based cooperation is the strongest, business-based cooperation has the widest scope, while proximity-based cooperation exists throughout these two main models of cooperation. The inter-regional cooperation level in China's COVID-19 patent cooperation network shows a decreasing trend from eastern to central to western regions. Eastern provinces and cities have stable partners; collaborative innovation in central provinces and cities need to be further improved; whereas there is still a lot of room for development in the western provinces and cities in establishing patent cooperation with other regions.

Coastal provinces and cities, such as Beijing, Shanghai, and Guangzhou, occupy a core position in the network, and play an important role in utilizing structural holes and bridging. Patent applicants with high centrality are mostly firms. Research institutes and universities mainly play the role of bridges, and research institutes and firms make greater use of structural holes.

From the perspective of subgroup structure, the number of external relationships of subgroup nodes at the province and city levels is significantly greater than that of internal relationships. There is no large number of cliques or consequent information barriers, indicating a great potential for development. In contrast, there is a tendency for cliques to develop at the applicant level. The common characteristics of subgroups at this level are that the cooperation is based on kinship and business and that proximity-based cooperation exists throughout these two main models of cooperation.

### Implications

The findings have the following implications.

First, efforts should be made to explore the potential for cooperation between patent applicants in the field of COVID-19. By developing technical information sharing platforms and incentive mechanisms, the low information transmission efficiency caused by technological distance can be overcome, and new cooperation can be established between patent applicants on an ongoing basis, thereby expanding the breadth of cooperation and exploring the potential for cooperation within the patent cooperation network.

Second, important nodes in the COVID-19 patent cooperation network should be encouraged to play a leading role. Accordingly, efforts should be made to promote the core nodes to carry out further patent cooperation, and to facilitate patent cooperation between the core and edge nodes under the leadership of the core nodes. In this way, the internal resource allocation of the network can be optimized, the sustainable development of the COVID-19 industry promoted, the bridging role of universities and research institutes further enhanced, and the resources of firms fully utilized.

Third and lastly, the government should subsidize the pharmaceutical R&D of relevant firms on a long-term basis and create technical reserves and strategic models for public health drugs to deal with large-scale public crises. Actions should be taken to establish a cross-regional cooperative group for COVID-19 patent development. Efforts should be made to fully exploit the technical advantages of industry-university-research organizations in coastal provinces and cities, promote the interconnection and interaction between the eastern and central regions, and strengthen the leading role of the east for the west, thereby achieving cross-regional COVID-19 cooperation and establishing a reasonable spatial pattern for promoting the COVID19 industry in China.

Despite its contribution, this study has some limitations. On the one hand, this study is limited to China, and did not investigate the COVID-19 patent cooperation between countries in the context of globalization. On the other hand, due to space limitations, the factors influencing China's COVID-19 patent cooperation network were not analyzed.

## Data availability statement

The original contributions presented in the study are included in the article/supplementary material, further inquiries can be directed to the corresponding author/s.

## Author contributions

WX contributed to conception and design of the study. JY and LQ organized the database and performed the statistical analysis. All authors contributed to manuscript revision, read, and approved the submitted version.

## Funding

This work was supported by the Major Program of the National Social Science Foundation of China (Grant Number 21&ZD139).

## Conflict of interest

The authors declare that the research was conducted in the absence of any commercial or financial relationships that could be construed as a potential conflict of interest.

## Publisher's note

All claims expressed in this article are solely those of the authors and do not necessarily represent those of their affiliated organizations, or those of the publisher, the editors and the reviewers. Any product that may be evaluated in this article, or claim that may be made by its manufacturer, is not guaranteed or endorsed by the publisher.

## Footnotes

^1^ https://xs.dailyheadlines.cc/scholar?start=10&q=medicine+Patent+Cooperation+Network&hl=zh-CN&as_sdt=0,5#:~:text=Wang%20L%2C%20Wang%20J%2C%20Yu%20W.%20The%20influence%20of%20patent%20cooperation%20network%20on%20growth%20of%20technology%2Dbased%20SMEs%3A%20an%20example%20of%20the%20pharmaceutical%20industry%20in%20China%5BM%5D//Innovative%20Capabilities%20and%20the%20Globalization%20of%20Chinese%20Firms.%20Edward%20Elgar%20Publishing%2C%202020
